# Cancerous Disease of the Stomach with Secondary Infection through the Thoracic Duct

**Published:** 1905-07-01

**Authors:** 


### CANCEROUS DISEASE OF THE STOMACH WITH SECONDARY INFECTION THROUGH THE THORACIC DUCT.

It is an important clinical memorandum that enlargement of the lymphatic glands at the root of the neck may be an index of malignant disease in the upper part of the alimentary canal. The significance of this rule is all the greater seeing that such enlargement may be the one fact which attracts the patient's attention, whilst dysphagia or other symptoms pointing to the primary growth may be unrecognised. Even a considerable epitheliomatous ulcer of the pharynx[1] has been known to cause no local complaint, and has only brought the patient under medical observation when there was secondary extension to the cervical glands. The value of the observation may also be illustrated in cases of dysphagia due to œsophageal obstruction. The fact of the obstruction may be determined beyond doubt by the use of a bougie, but there remains the important question, What is the cause of the obstruction? and in such circumstances cervical glandular enlargement becomes of high clinical importance. In this association considerable interest is attached to a case of "latent" carcinoma of the stomach reported by Dr. W. Mitchell Stevens,[2] in which an early diagnosis was made from the existence of glandular enlargement in the left supra-clavicular region. The patient, a man of 46 years, presented himself at hospital with œdema of the genitals and legs of about a week's duration. On physical examination the only additional abnormal facts discovered were a moderate painless enlargement of the liver and an enlarged gland under the clavicular insertion of the left sterno-mastoid muscle. The gland was fairly movable but very hard. There was no evidence of disease in the heart, lungs, or kidneys, and the blood gave evidence of a "secondary" anæmia. From these facts it was argued that in the absence of evidence of disease in the neighbourhood the cervical enlargement was probably the result of malignant disease within the abdomen where, as physical examination showed, the liver was unduly large. Further, as malignant disease of the liver is usually secondary, it was suggested, as no symptoms or evidence of a primary growth could be detected, that the case was one of latent carcinoma of the stomach, and that the œdema was probably due to the pressure of enlarged (malignant) glands on the large venous trunks within the abdomen. The course of the case appeared to justify the accuracy of these conclusions for the hepatic and glandular enlargements continued to increase and the patient gradually to lose flesh and strength; later, too, gastric pain, vomiting and hæmatemesis were added to the symptoms. At the autopsy the case was found to be one of malignant disease of the stomach, the growth involving the posterior wall, and the condition of the liver and the cervical glands was in exact harmony with the original diagnosis. An interesting demonstration that the route of extension to the cervical glands was by the thoracic duct was afforded by the extensive malignant involvement of the wall of the duct. The sections of the gland showed a structure identical with the gastric tumour. Dr. Nathan Raw[3] records a somewhat similar case in which the primary malignant process was in the pancreas. The patient, a man of 46 years, complained only of general weakness and of a swelling in the left side of the neck. No evidence of disease could be found in any other part of the body, and the condition was regarded as lymphadenoma. Gradually the patient got weaker and ultimately died, when the pancreas, the abdominal glands, and the cervical mass were found to be the sites of scirrhous growth. Here also the lower end of the thoracic duct was involved in the disease. These cases obviously have an important bearing on the discussion regarding the mode in which cancer spreads, and will greatly strengthen the position of those who claim that the thoracic duct plays an important part in the process. Possibly, as Dr. Raw suggests, a careful examination of the thoracic duct in cases of death with apparently primary malignant disease of the cervical lymphatic glands would trace not a few of these to a "latent" malignant growth in the abdomen. In the field of diagnosis, too, these records emphasise the importance of considering carefully the possible meaning of a cervical glandular enlargement. Occurring without explanation, such an event demands the most anxious study of the possibility of malignant disease in some part of the alimentary canal.

[1] Polyclinic Jour., vol. v., p. 290. [2] Brit. Med. Jour., April 29, 1905. [3] Ibid., June 24, 1905.

